# Newly Developed Prodrugs and Prodrugs in Development; an Insight of the Recent Years

**DOI:** 10.3390/molecules25040884

**Published:** 2020-02-17

**Authors:** Anas Najjar, Abderrahman Najjar, Rafik Karaman

**Affiliations:** 1 Faculty of Pharmacy, Department of Bioorganic & Pharmaceutical Chemistry, Al-Quds University, Jerusalem P.O. Box 20002, Palestine; nash.najjar@gmail.com; 2 Institute of Pathology, Rabin Medical Centre, PetachTikva 49100, Israel; abderrahman.najjar@gmail.com

**Keywords:** ACT-281959, ANAVEX 2-73, baloxavir marboxil, clinical trial, evofosfamide, fostemsavir, ixazomib, pretomanid, prodrug, selexipag

## Abstract

Background: The design and development of prodrugs is the most common and effective strategy to overcome pharmacokinetic and pharmacodynamic drawbacks of active drugs. A respected number of prodrugs have been reached the drugs market throughout history and the recent years have witnessed a significant increase in the use of prodrugs as a replacement of their parent drugs for an efficient treatment of various ailment. Methods: A Scan conducted to find recent approved prodrugs and prodrugs in development. Results: Selected prodrugs were reported and categorized in accordance to their target systems. Conclusions: the prodrug approach has shown many successes and still remains a viable and effective approach to deliver new active agents. This conclusion is supported by the recent approved prodrugs and the scan of clinical trials conducted between 2013–2018.

## 1. Introduction

Prodrugs are biologically inactive compounds that are activated post-administration to their pharmacologically active forms. Often prodrugs are formulated to overcome pharmacokinetic barriers such as poor solubility and absorption, extensive first-pass metabolism, or rapid excretion, and pharmacodynamic barriers such as toxicity, side effects, and poor efficacy. The activation of prodrugs is usually *via* either enzymatic processes such as that by cytochrome enzymes, esterases and amidases or chemical processes (inter or intra-molecular) such as hydrolysis and oxidation.

Many prodrugs have enjoyed clinical success in treating various chronic and acute conditions [1]. Among the successful examples are the prodrugs indented for the management of hypertension such as the angiotensin-converting enzyme inhibitors (ACEIs) and angiotensin receptor blockers (ARBs). Others are those used to inhibit platelet aggregation in the cases of clotting disorders and cardiac incidents such as clopidogrel and prasugrel. Sulfasalazine is a common choice prodrug for the management of ulcerative colitis and Crohn’s disease.

Although the production of biological treatments such as monoclonal antibodies is considered as a promising strategy to invent new medicines, the prodrug approach is still being explored and novel prodrugs are still being developed. During the years 2008–2017, 12.4% of all new molecular entities approved by the FDA were prodrugs (31 out of 249) [2].

However, one might wonder whether recent clinical trials reflect the future of prodrugs as new treatments, parts of combined treatment regimens, or treatments for new indications other than their already approved ones. In this review, a summary of selected prodrugs in clinical trials during the years 2013–2018 is reported.

## 2. Methodology

A scan of the clinical trials (NCTs) database at clinicaltrials.gov was carried out at the end of 2018 for clinical trials first posted after the 1st of January 2013, encompassing 5 years of clinical trials. The status of the clinical trial was not taken into consideration as all trials that were either recruiting or not, active, completed, suspended, etc… were all included in the scan. This returned 153,851 unclean results.

Filters were applied to clean the results as follows:

(1) Firstly, clinical trials that included ‘drug’, ‘combination’, or ‘biological’ as their intervention were selected returning 54,895 interventions that included duplicate NCTs. This is due to trials using different interventions.
(2) Secondly, in accordance with NCT number and intervention, duplicates were removed to only include unique interventions returning 25,844 unique interventions.
(3) Thirdly, a further filter was applied to include only the intervention itself without regard to dosage, administration route, combination type, or regimen. This produced 12,364 unique interventions.
(4) Finally, Prodrugs were then identified with the aid of previous lists of prodrugs as well as research into each intervention that was not previously listed as a prodrug.

Herein, the trend and results of clinical trials on selected prodrugs during the aforementioned period are reported. The prodrugs were sorted in accordance with the physiological systems they treat or being studied to treat.

## 3. Selected Prodrugs

### 3.1. Cardiovascular System

#### 3.1.1. Simvastatin

**Figure 1:**
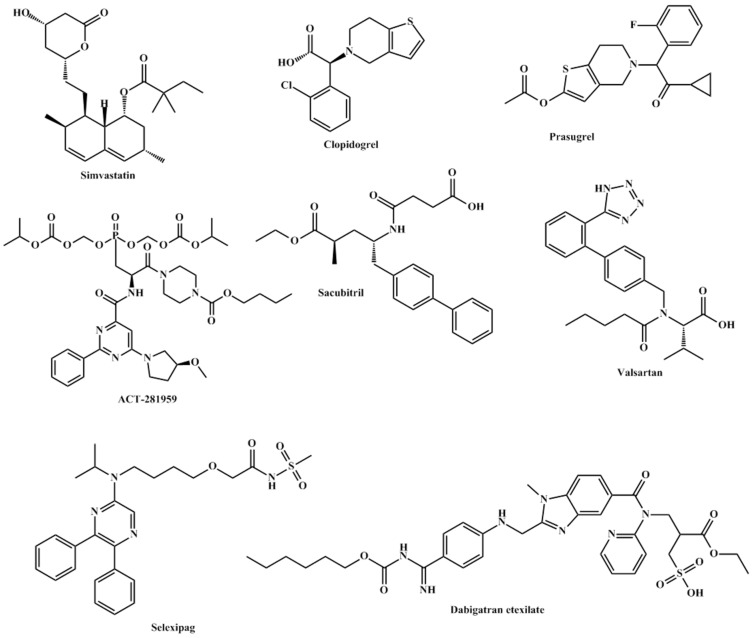
Structures of prodrugs reported in this sub-section.

Simvastatin (Figure 1) is amongst the oldest and best-known prodrug available on the market. Its mechanism of action involves in vivo hydrolysis of its 6-membered lactone ring to yield the beta, delta-dihydroxy acid, and an active metabolite that is similarin structure to HMG-CoA (hydroxymethylglutaryl CoA). The hydrolysis metabolite of simvastatin competes with HMG-CoA for HMG-CoA reductase, which catalyzes the transformation of HMG-CoA to mevalonate, a rate-limiting step in cholesterol biosynthesis. However, many clinical trials were investigating the effects of statins in combination or as sole treatments during 2013–2018. The majority of those trials focused on the interactions between simvastatin and conditions or diseases assessing its safety and preferability over other statins under certain conditions. On the other hand, some trials used only simvastatin in their studies and have yet to post results. These include NCT03011931 which used simvastatin metabolism as a test for celiac disease activity, NCT03131726 which studied the efficacy of simvastatin in the treatment of Graves’ Opthalmopathy and NCT03387670 which is a phase 3 trial of simvastatin in multiple sclerosis titled MS-STAT2. The latter was conducted following the results of MS-STAT1 which showed that loss of neurons is reduced in patients receiving simvastatin compared to placebo [1,2].

The secondary progressive MS stage (SPMS) is the cause of the great disability in patients with MS. Till today, there are very few medicines that can treat effectively or slow the disability progression in patients with SPMS. The MS-STAT2 trial establishes that the prodrug simvastatin currently used to treat vascular disease and high cholesterol levels could be utilized as an effective therapeutic for the treatment of SPMS. This is due to the potential immunomodulatory and neuroprotective properties of the prodrug.

#### 3.1.2. Clopidogrel and Prasugrel

**Scheme 1:**
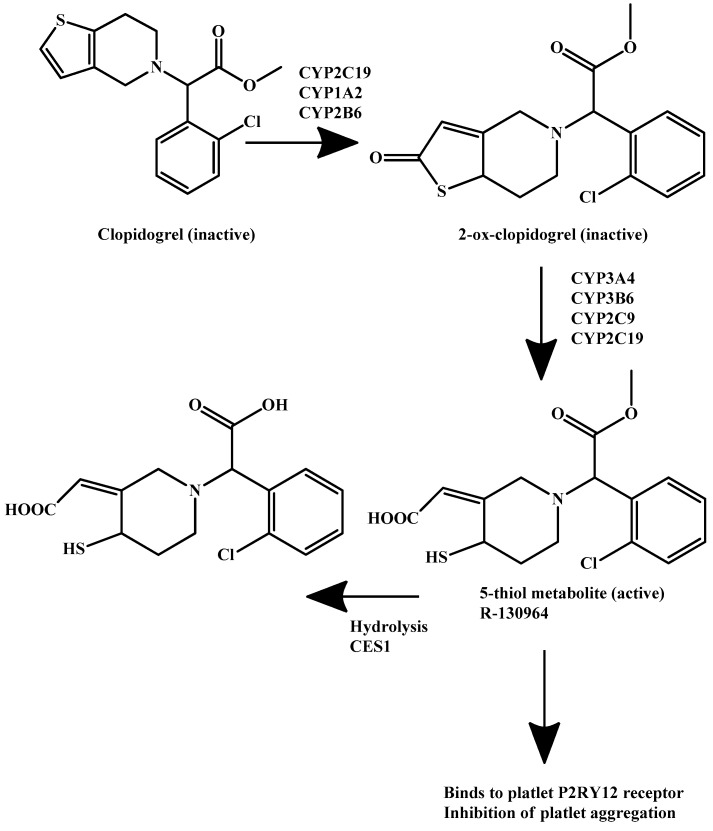
Metabolic pathway and mechanism of action of Clopidogrel.

Adenosine diphosphate receptor blockers, especially thienopyridines, are proven excellent platelet aggregation inhibitors and remain of the first choices in the prevention of clotting disorders and follow up treatment of cardiovascular incidents. Platelet inhibition of this class of drugs is mainly achieved through blocking P2Y12 receptors of platelets. Furthermore, studies reported that clopidogrel (Figure 1) inhibits collagen and thrombin-induced platelet aggregation (for activation pathway and mechanism of action of clopidogrel see Scheme 1) [3].The majority of the recent clinical trials on clopidogrel and prasugrel (Figure 1)were to further establish the best doses of the drugs, their regimens and their interactions with other common chronic conditions such as diabetes. However, no new indications were being explored. Thus, it is expected that those recent clinical trials will aid in the development of future guidelines for conditions such as acute coronary syndrome, angina, heart failure, atrial fibrillation, and others.

It is worth noting that careful consideration should be taken into consideration in the trials with clopidogrel and prasugrel prodrugs when given with other medicines intended to treat other ailments. These medicines have the potential to interfere with the prodrugs’ activation of the prodrug resulting in reversing the patient’s recovery. 

#### 3.1.3. ACT-281959

In contrast to thienopyridines such as clopidogrel and prasugrel, ACT-24647 (Figure 1) is a novel potent P2Y12 receptor inhibitor that is being developed for early intervention through subcutaneous injection. ACT-281959 is the oral prodrug of ACT-24647 which is activated through hydrolysis of the two ester chains on its phosphonic acid [4]. One clinical trial coded NCT01954615was conducted to evaluate the safety, tolerability, pharmacokinetics, and pharmacodynamics of ACT-281959. The trial revealed that the prodrug is well-tolerated, produced dose-dependent concentrations of the active form. The only frequent side effect was headache and no bleeding or dyspnea cases were reported. The prodrug merits further investigation in patients with coronary artery disease [5].

#### 3.1.4. Sacubitril and Valsartan

Sacubitril (Figure 1) is a prodrug of LBQ657, a neprilysin inhibitor, which was approved in 2015. Neprilysin is an endopeptidase responsible for cleaving atrial, brain, and c-type natriuretic peptides which normally produce vasodilation, natriuresis, and diuresis [6]. However, since sacubitril is administered in combination with valsartan, vasodilation and decreased vascular resistance are produced due to the accumulation of natriuretic peptides and inhibition of angiotensin II. Sacubitril is generally used in combination with valsartan (Figure 1) in the Entresto drug.

Current research focuses on the safety and efficacy of the aforementioned prodrugs. Newer research explores the use of the combination in the presence of comorbidities such as thyroid cancer, breast cancer, and diabetes. Post marketing research is being focused on understanding the exact mechanism of action of this combination.

The clinical trials with this combination in the presence or absence of comorbidities should take into consideration that almost all cardiovascular diseases are a result of many contributing factors.

#### 3.1.5. Selexipag

Selexipag (Figure 1) was granted approval by the FDA in 2015 for the treatment of pulmonary arterial hypertension. Along with its active metabolite ACT-333679, they are prostacyclin receptor agonists which increase pulmonary circulation vasodilation and consequently decrease pulmonary arterial hypertension [7]. During 2013-8 sixteen clinical studies were reported for selexipag. In healthy subjects, bioavailability NCT02882425, dose-response NCT02745860, interactions with clopidogrel NCT03496506 along with drug-drug interactions with gemfibrozil and rifampicin NCT02770222 were all tested. Current trials focus on the use of selexipag in children with pulmonary arterial hypertension NCT03492177, and patients with chronic thromboembolic pulmonary hypertension NCT03689244. Preliminary results of trials which have published the outcomes of their trials show that selexipag is tolerable and pharmacokinetically and clinically effective [8].

#### 3.1.6. Dabigatran Etexilate

Dabigatran etexilate (Figure 1) is a synthetic reversible direct inhibitor of thrombin. This inhibition results in decreased amounts of fibrin leading to disruption of the clotting process. Dabigatran is activated by the liver and plasma esterases through hydrolysis to yield its active form. The advantage of using this prodrug over other drugs like warfarin is that continuous lab monitoring is not needed [9]. Current observations still show a relatively low number of patients receiving dabigatran. This is due to the availability of newer more potent agents such as oral factor Xa inhibitors have shown a decrease in the use of dabigatran in the clinic. 

### 3.2. Nervous System

#### 3.2.1. ANAVEX 2-73

ANAVEX 2-73 or Blarcamesine is a small molecule orphan drug, developed by Anavex Life Sciences Corp., which activates sigma-1 receptors in neurons. This activation modulates processes related to neurodegeneration by preventing or decreasing protein misfolding, cellular stress, mitochondrial dysfunction, and oxidative stress [10]. ANAVEX 2-73 is an aminotetrahydrofuran which is activated through demethylation of its tertiary amine group [11].

**Figure 2:**
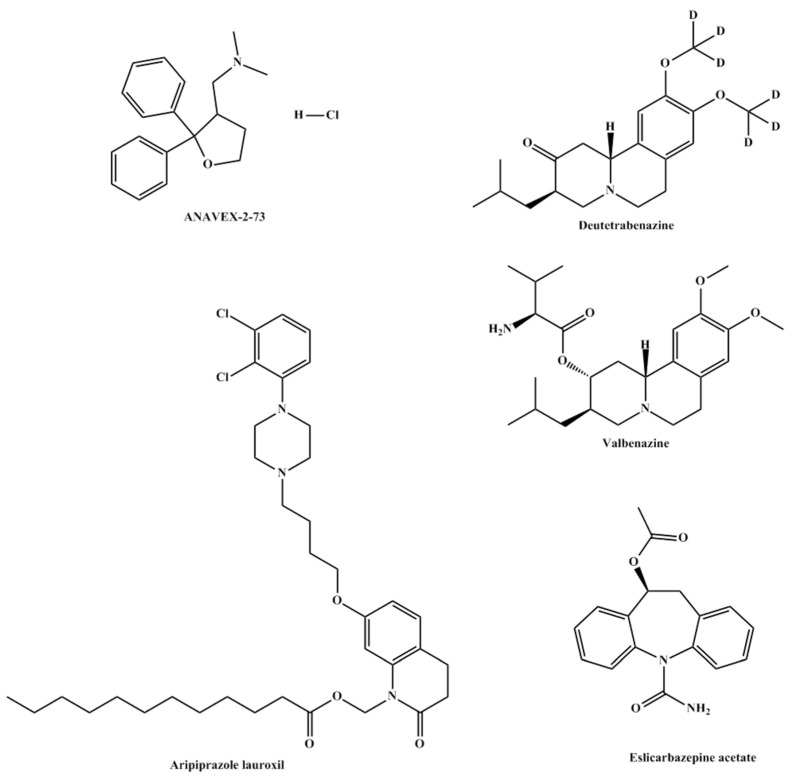
Structure of prodrugs reported in this sub-section.

The main diseases ANAVEX 2-73 (Figure 2) is being tested on are Rett Syndrome and Alzheimer’s disease. NCT03758924, also identified as ANAVEX2-73-RS-001, is the only FDA approved phase 2 trial. While results are scarce and limited to the manufacturing corporation’s website, this drug appears to be promising, either in its treatment potential or its potential as a lead compound from which better sigma receptor agonists can be inspired.

#### 3.2.2. Valbenazine and Deutetrabenazine

Valbenazine (Figure 2) prodrugis the L-valine ester of [+]-α-dihydrotetrabenazine (DTBZ) which undergoes hydrolysis in a fast manner to its active drug, DTBZ. Valbenazine was developed under the name NBI-98854 and was approved by the FDA for the treatment of Tardive Dyskinesia in 2017. Valbenazine’s mechanism of action is mediated through the reversible inhibition of VMAT2 in the treatment of TD. VMAT2 is selective to the central nervous system and is responsible for the transport and recycling of neurotransmitters across the synapse. The inhibition of VMAT2 augments neurotransmitter degradation and results in presynaptic neurotransmitter depletion, particularly of dopamine. Both valbenazine and its active metabolite DTBZare active inhibitors of vesicular monoamine transporter 2. Similarly, deutetrabenazine (Figure 2) was also approved in 2017 and is also metabolized to *a*-dihydrotetrabenazine. Both prodrugs allowed for once-daily dosing due to decreased hepatic metabolism and demonstrated high selectivity for vesicular monoamine transporter 2 [12]. This inhibition results in a decreased uptake of neurotransmitters, mainly dopamine. Patients having tardive dyskinesia and Parkinson’s disease show a decreased number of dopaminergic neurons. The inhibition allows for higher concentrations of dopamine in the neuron synapses leading to a decrease in symptoms [13].

Several recent clinical trials investigated the safety and efficacy of both prodrugs in the treatment of chorea and Tourette syndrome. Results are yet to be published but timely completion of the trials appears to be promising.

#### 3.2.3. Aripiprazole Lauroxil

Aripirazole lauroxil (Figure 2) is a long-acting injectable prodrug of aripiprazole which is indicated for the management of schizophrenia and bipolar disorder [14,15]. Following intramuscular injection, the prodrug is hydrolyzed to form N-hydroxymethyl-aripiprazole which, in turn, undergoes spontaneous cleavage to aripiprazole. The mechanism of action of the active metabolite is the agonism of dopaminic and 5-HT1A receptors as well as alpha-adrenergic 5-HT2A receptors [16]. However, and while the binding profile of aripiprazole is known, how it exerts its antipsychotic activity is not well established yet. However, side effects such as orthostatic hypertension are linked to agonism of the alpha-adrenergic receptor.

The main advantage of the prodrug is that it represents a sustained release dosage form of the active drug. This results in better adherence in patients who have difficulty adhering to their medications [17].

#### 3.2.4. Eslicarbazepine Acetate

Eslicarbazepine acetate prodrug (Figure 2) undergoes hydrolysis during first-pass metabolism to yield eslicarbazepine. The metabolite, eslicarbazepine, is an anticonvulsant used for partial-onset seizures in epilepsy patients. The mechanism of action of the metabolite is not yet been well understood. Studies showed that the prodrug produces modest activity with mild side effects.

The main strategy behind this prodrug is to avoid the formation of eslicarbazepine epoxide before reaching the systemic circulation [18,19].

Precautions should be taken when using this prodrug since its administration might lead to suicidal events.

### 3.3. Oncology

#### 3.3.1. Ixazomib

**Figure 3:**
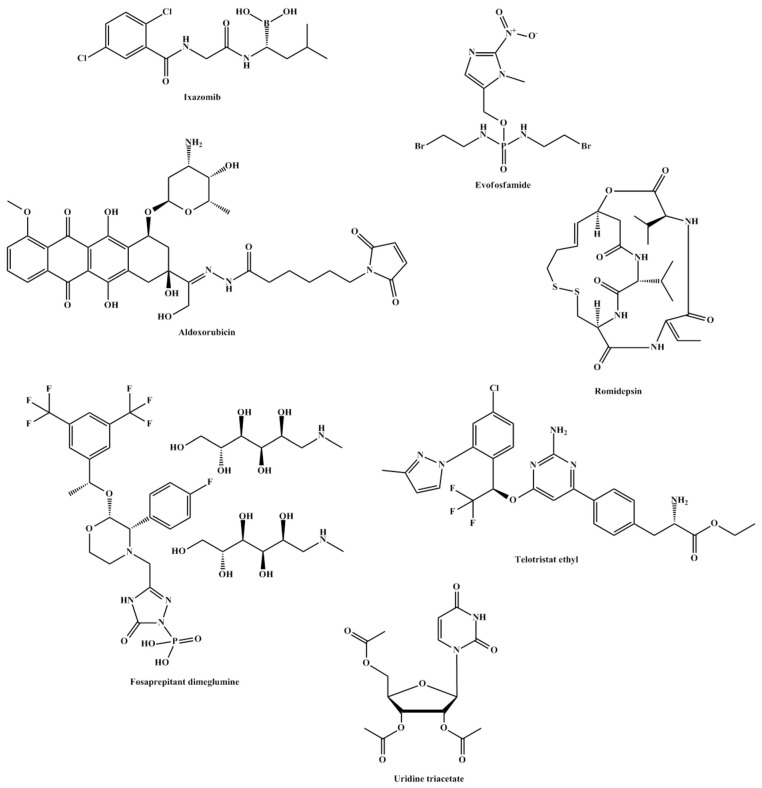
Structures of prodrugs reported in this sub-section.

The ester prodrug of Ixazomib, ixazomib citrate (Figure 3) is used in the cases of multiple myeloma. The prodrug undergoes into its parent drug via hydrolysis. Ixazomib’s mechanism of action involves a reversible inhibition of the beta 5 subunit of the 20S proteasome. Ixazomib was first approved by the FDA in 2015 in combination with lenalidomide and dexamethasone. It is currently marketed by Takeda Pharmaceuticals under the brand name Ninlaro® as ixazomib citrate. A total of 34 NCTs studied ixazomib as a sole therapy or in combination during the start of 2013 till the end of 2018. The earliest NCTs revolved around the pharmacokinetics, safety, efficacy, and tolerability of ixazomibin mainly multiple myeloma patients in 2011–2012. Newer NCTs are now focused on the effect of ixazomib in multiple sclerosis, lymphoma, sarcoma, and leukaemia.

In a phase 1 study by Takeda Pharmaceuticals (NCT01830816) the pharmacokinetics and safety of ixazomib were evaluated in patients with relapsed/refractory multiple myeloma and advanced solid tumors in light of renal function. The study published in June of 2019, showed that ixazomib was less tolerated in patients with decreased renal function with an increase in the adverse effects.

In a randomized phase 2 study [20], NCT02046070 a combination therapy of ixazomib plus cyclophosphamide and low-dose dexamethasone was evaluated in transplant-ineligible patients. The study revealed that this treatment regimen is tolerable with manageable toxicity. Furthermore, toxicity rates were reported more in patients receiving 400 mg/m^2^ of cyclophosphamide in comparison to those who received 300 mg/m^2^ of the combination suggesting the latter dose to be more tolerable.

Currently, the following are selected trials in progress aiming to study ixazomib in patients with peripheral T-Cell Lymphoma (NCT03547700), Mantle Cell Lymphoma (NCT04047797 and NCT03616782), B-Cell Lymphoma (NCT02898259), HIV (NCT02946047), and multiple myeloma (NCT03608501 and NCT03770260) and Triple-Negative Breast Cancer (NCT02993094).

#### 3.3.2. Evofosfamide

Evofosfamide, also known as TH-302(Figure 3) is a hypoxia-activated prodrug of brominated isophosphoramide mustrd. The active form is a potent DNA alkylator. TH-302 is being tested for efficacy in a variety of cancers such as pancreatic, oesophageal, soft tissue sarcoma, and solid tumors. However, many of the trials were terminated due to the lack of enrolment, lack of efficacy and failure to meet endpoints. 

However, newly published studies still find advantages and great hope in proceeding with the prodrug [21,22,23]. This contradiction in reports could open the door in further investigation of the drug or the strategy of hypoxia-activated prodrugs.

#### 3.3.3. Aldoxorubicin

In sarcoma therapy, anthracyclines in general and doxorubicin, in particular, remain cornerstones. However, their dose-dependent adverse effects and significant toxicities, especially cardiac toxicity, greatly limit their potential use [24]. The development of aldoxorubicin (Figure 3) by conjugating doxorubicin to albumin allowed for lower plasma concentrations of doxorubicin and thus leading to fewer side effects. The conjugate accumulates in tumor cells and is then cleaved by liposomes to doxorubicin and albumin. Studies have shown that due to decreased side effects higher doses can be administered allowing for stronger tumor inhibition [25].

While the first human trial of aldoxorubicin was reported in 2006 [26], aldoxorubicin is being used as part of a regimen of drugs rather than as sole therapy in sarcomas. However, a scan of clinical trial testing for aldoxorubicin showed that many of the trials were not sufficiently well designed due to the highly diverse term “sarcoma”. 

It is worth mentioning that older preclinical data demonstrated the superiority of aldoxorubicin over doxorubicin at least in the toxicity profile. Therefore, one can assume that as long as doxorubicin remains one of the preferred treatment options, then aldoxorubicin merits further better structured clinical trials to prove its potential as a better replacement to doxocorubicin.

#### 3.3.4. Fosaprepitant Dimeglumine

Fosaprepitant dimeglumine (Figure 3) is a prodrug of aprepitant. It is dephosphorylated by phosphatase to its active form. Phospohorylation of aprepitant leads to increased aqueous solubility of aprepitant, a strategy used in prodrug design to overcome solubility issues. It is indicated for the treatment and mainly prevention of chemotherapy-induced emesis. The prodrug is available in an injectable IV form which poses a great advantage for patients suffering from continued vomiting. Studies showed that a one-day regiment of the prodrug is equal to a 3-day regimen of aprepitant, the common regimen used today [27].

The superiority of this prodrug over its parent drug stems from the higher aqueous solubility of the prodrug leading to better bioavailability and a more efficient clinical profile. 

#### 3.3.5. Romidepsin

Romidepsin (Figure 3) is a prodrug indicated for the treatment and management of peripheral T-cell lymphoma (PTCL). It is activated by intracellular glutathione yielding a metabolite with a free thiol group. The metabolite is a potent and selective inhibitor of histone deacetylase. This inhibition results in enhanced histone acetylation which influences the cell cycle, leading to apoptosis. 

Although patients with PTCL are typically receiving aggressive first-line chemotherapy they suffer inadequate responses and poor prognosis. The prodrug, romidepsin is considered as a single-agent therapy that provides durable responses in patients with refractory or relapsed/PTCL.

Studies have shown a synergistic effect when romidepsin and pralatrexate were used in a combination with tolerable hematologic toxicity. These studies and other suggest additional PTCL indications for romidepsin. Others studies indicated that the use of romidepsin with other antineoplastic agents further improved the drug response [28].

#### 3.3.6. Telotristat Ethyl

In 2017 telotristat ethyl (Figure 3) was approved for the treatment of diarrhea associated with caracinoid syndrome [29]. The treatment encompasses the prodrug in combination with somatostatin. In carcinoid syndrome serotonin levels are increased, leading to symptoms such as diarrhea. The prodrug is activated by carboxylesterase to Lp-778902. The active form decreases serotonin levels throughout the gastrointestinal tract by selective inhibition of tryptophan hydroxylase [30].

#### 3.3.7. Uridine Triacetate

Uridine triacetate (Figure 3) is the acylated prodrug of uridine. It is metabolized by esterases to yield active uridine. It is used as an antidote for fluorouracil and capecitabine overdose. Capecitabine is a prodrug of fluorouracil that inhibits methylation of deoxyuridic acid to thymidylic acid [31]. This leads to rapidly occurring toxicity caused by impaired clearance of fluorouracil due to dihydropyrimidine dehydrogenase deficiency or genetic variations in the enzymes which metabolize fluorouracil.

The prodrug, uridine triacetate, has been reported to deliver 4 to 6 fold more uridine to systemic circulation than equal equimolar doses of uridine alone [32]. This indicates that the prodrug is more bioavailable and effective than its parent drug. This might be due to the slow hydrolysis of the prodrug to its active metabolite.

### 3.4. Antivirals

#### 3.4.1. Baloxavir Marboxil

**Figure 4:**
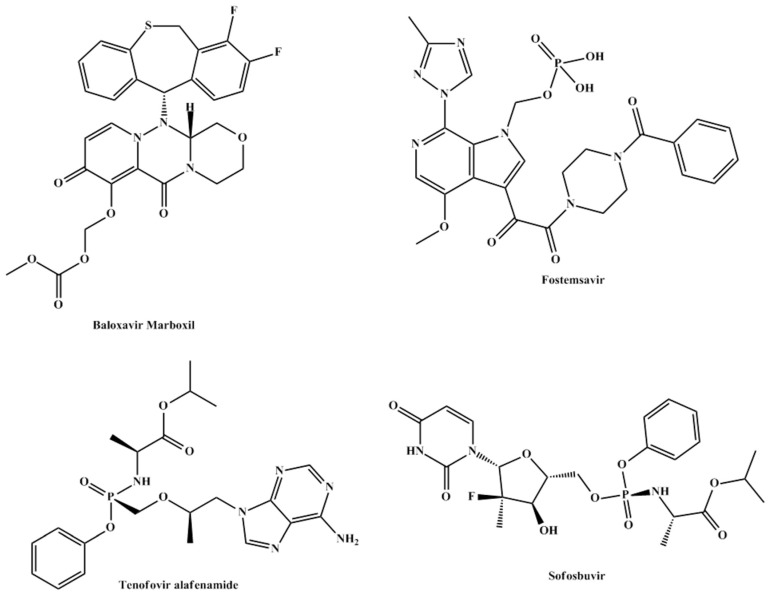
Structures of prodrugs reported in this sub-section.

Baloxavir marboxil (Figure 4) is a prodrug that is hydrolyzed to its active metabolite, baloxavir. Being the first new antiviral agent for influenza in nearly 20 years, baloxavir marboxil made headlines following its approval in 2018.Themechanism of action of the drug, baloxavir, is *via* inhibition of CAP endonuclease [33].

The prodrug is administered in the first 48 hours following symptoms of influenza and decreases viral shedding by inhibiting viral CAP endonuclease. During 2013–2018 only 5 clinical trials reported the prodrug in their intervention three of which have been completed and were aimed at comparing it to placebo and oseltamivir and assessing its safety and efficacy. Currently, baloxavir marboxil is indicated for patients over the age of 12 but one clinical trial NCT03653364was aimed to assess the safety and efficacy of the treatment in infants less than 1-year-old. If the results of this trial are positive, the drug could be indicated for younger patients signaling a better and narrower epidemiological future of influenza worldwide.

#### 3.4.2. BMS-663068 or Fostemsavir

The phosphonooxymethyl prodrug of temsavir (BMS-626529) known as fostemsavir or BMS-663068 (Figure 4) has a unique mechanism of action that involves binding to the envelope glycoprotein 120 of HIV resulting in the prevention of viral attachment to the host CD4 cell surface receptor. 

In a phase 2b study of treatment-experienced individuals, fostemsavir appeared to be well tolerated. Phase 3 studies are ongoing [34]. The safety and efficacy of the drug were established in AI438011, a phase 2b randomized controlled trial, and was found to be well tolerated in the majority of tested patients [35]. During 2013–2018, additional 15 clinical trials were carried out to assess the efficacy, pharmacokinetics, interactions, and toxicity of the prodrug. All trials supported a continuation of the testing of the prodrug and currently an ongoing phase 3 trial (BRIGHTE or NCT02362503) is showing promise with final results expected to be posted in 2024.

If the results of the trial are positive, the drug could signal a new era in HIV-1 treatment especially in heavily treated patients in which the virus has developed significant resistance to classical therapy [36].

#### 3.4.3. Tenofovir Alafenamide

Tenofovir alafenamide (Figure 4) is an acyclic analog of dAMP; it is phosphorylated to its active form, tenofovir diphosphate, by AMP kinase. The active form of the prodrug inhibits HIV reverse transcriptase [37]. Currently, tenofovir alafenamide is indicated for the treatment of chronic hepatitis B in patients with compensated liver disease [38] and HIV-1 infections in combination with emtricitabine. 

When compared to its prodrug counterpart tenofovir disoproxil, tenofovir alafenamide has been shown to produce lower systemic levels and higher intracellular levels producing better delivery and potency [39].

#### 3.4.4. Sofosbuvir

**Scheme 2:**
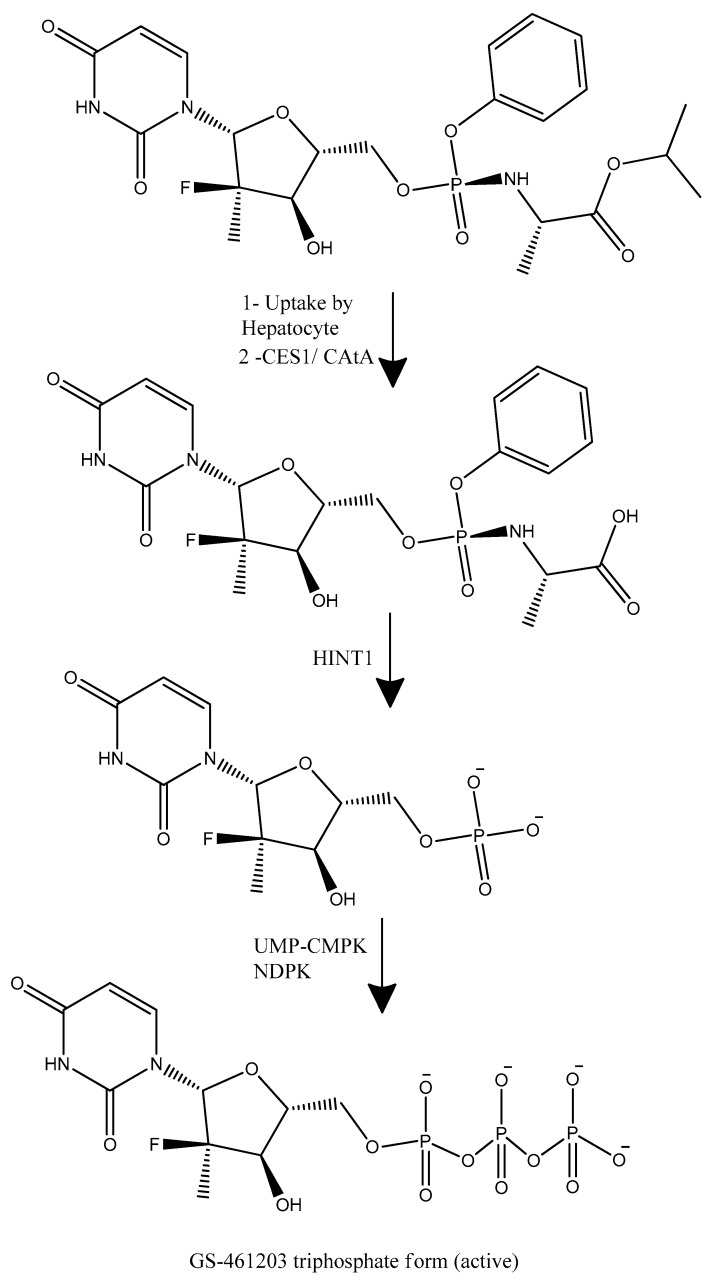
The activation pathway of sofosbuvir in hepatocytes.

Sofoxbuvir (Figure 4) is a prodrug indicated for the treatment of hepatitis C infection. It undergoes 3 steps intracellular activation by cathepsin-acarboxylase 1, histidine triad nucleotide-binding protein 1, and uridine monophosphate cytidine monophosphate kinase. The three-step activation pathway yields GS-461203, the active form of the drug (Scheme 2) [40]. 

The American Association for the Study of Liver Diseases recommended sofosbuvir as first-line therapy in the treatment of hepatitis C in 2016 [41]. Sofosbuvir is now more commonly prescribed as part of combination therapy with velpatasvir, ledipasvir or ribavirin. The studies on these combinations revealed well toleration to the regimen, fewer side effects, and decreased discontinuation rates. 

### 3.5. Tuberculosis, Malaria, and Bacterial Infections

**Figure 5:**
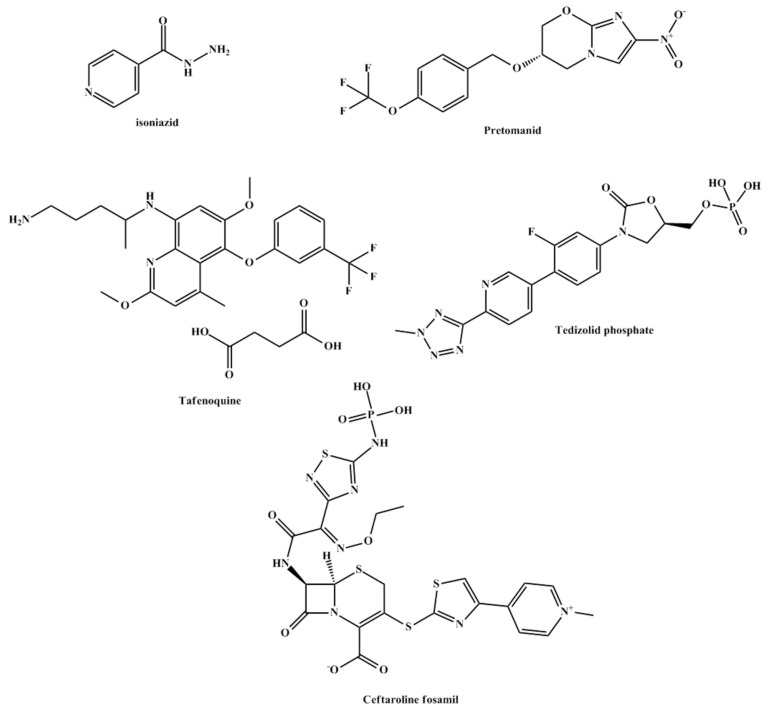
Structures of prodrugs reported in this sub-section.

The age-old treatment of choice for tuberculosis is isoniazid (Figure 5), one of the first prodrugs to be marketed. It is activated intracellularly by bacterial catalase to form an oxyferrous enzyme complex which leads to the inhibition of mycolic acid synthesis and thus, disruption of the bacterial cell wall [42]. In *Mycobacterium tuberculosis* isoniazid specifically inhibits enoyl reductase. Only one recent clinical trial (NCT03057756) reported isoniazid as part of combination therapy including Kanamycine, Moxifloxacine, Prothionamide, Isoniazide, Clofazimine, Ethambutol, and Pyrazinamide.

However, pretomanid (Figure 5), a new anti-tuberculosis drug has been developed to be administered in combination with linezolid and bedaquiline. This combination proved more efficacious in resistant tuberculosis in addition to requiring a shorter duration of treatment in contrast to the regimen in the aforementioned paragraph. Pretomanid was approved by the FDA in August 2019 [43] following several successful clinical trials showing good pharmacokinetic properties in healthy subjects, and combination therapy. Pretomanid exerts its bactericidal activity by increasing nitric oxide levels following its reduction to a desnitro derivative [44].

#### 3.5.1. Tafenoquine

Tafenoquine (Figure 5) is a recently approved prodrug for the treatment of malaria. It is converted to its active form, 5,6 ortho-quinone tafenoquine, by CYP2D6 [45]. The metabolite is taken up by the parasite and reduced to radicals intracellularly producing toxicity and parasite death. Patients who are born with G6PD deficiency are at high risk of haemolysis which limits the wide use of this prodrug [46].

#### 3.5.2. Tedizolid Phosphate

Tedizolid phosphate (Figure 5) is a prodrug of tedizolid which is indicated for the treatment of acute bacterial skin infections [47,48]. Tedizolid phosphate is converted by plasma phosphatases to its active parent drug, tidezolide. The latter has shown potent *in-vitro* activity against gram-positive bacteria including methicillin-resistant S. aureus [49].

The prodrug is more efficient than its parent drug due to its higher aqueous solubility and slow rate of dephosphoralytion to tidezolide leading to once-daily dosing with lesser side effects.

#### 3.5.3. Ceftaroline Fosamil

Similarly, ceftaroline fosamil (Figure 5), an older prodrug, is phosphorylated to improve aqueous solubility and is also activated by plasma phosphatase to yield ceftaroline. It is indicated for the treatment of acute bacterial skin and skin structure infections, as well as community- acquired pneumonia [50,51].

The advantage of the prodrug over its parent drug is due to the higher aqueous solubility of the prodrug which results in increased bioavailability and more efficient clinical profile.

### 3.6. Opthalmology

#### Latanoprostene Bunod

**Figure 6:**
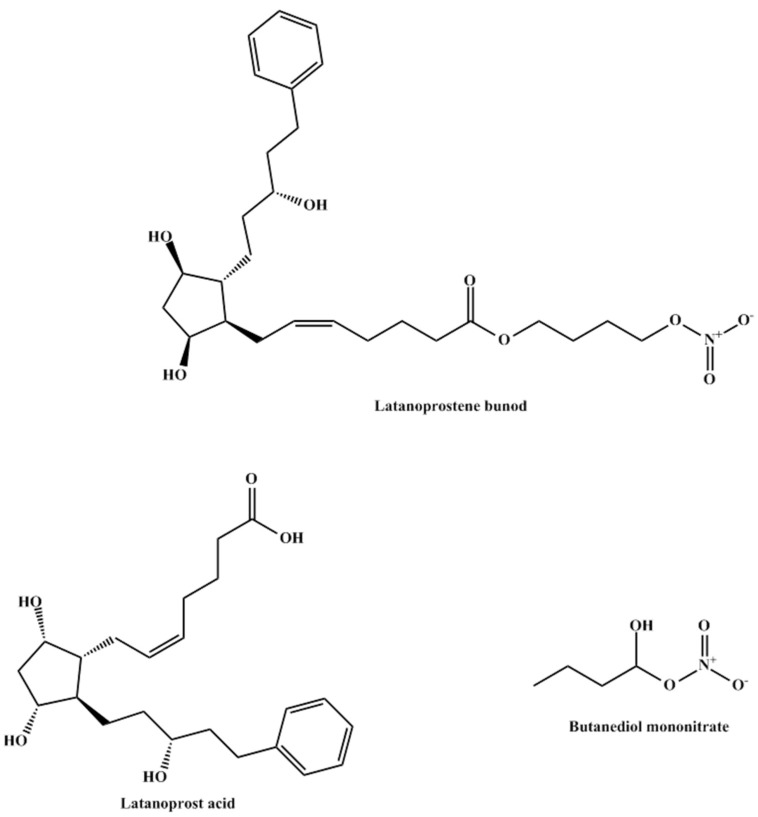
Structures of latanoprostene bunod, latanoprost acid, and butanediol mononitrate.

Latanoprostene bunod (Figure 6) is a prodrug of two active entities, latanoprost acid (Figure 6) and butanediol mononitrate (Figure 6), which yields NO, delivering them at a ratio of 1:1. The prodrug is hydrolyzed by corneal esterase yielding active agents. It is indicated for the treatment of glaucoma as both active agents reduce intraocular pressure [52,53].

Latanoprostene bunod enjoys a novel dual mechanism of action stemming from its ability to yield NO and prostaglandin F2-alpha analog latanoprost acid metabolite resulting in tissue and cell relaxation.

## 4. Summary

The period spanning from 2013 to 2018 witnessed the release of a large number of newly approved prodrugs. Some of the prodrugs were novel classes such as sacubitril, and some were ground breaking such as baloxavir marboxil.

Existing older prodrugs such as simvastatin, clopidogrel, and prasugrel are still being tested in clinical trials despite their well-established role in the clinic. Their clinical trials aim mainly towards the optimization of regimens, exploration of other indications, as well as administration in patients with other chronic illnesses.

Newer promising prodrugs such as fostemsavir, which is yet to gain approval, hold promise for patients with advanced HIV-1 infections. Furthermore, ANAVEX 2-73 could be the first in a new class of sigma receptor agonists for the treatment of Rett syndrome and Alzheimer’s disease. The prodrug appears to show promise and could lead to the production of a whole different class of drugs.

## 5. Conclusions and Remarks 

The failure of a respected number of drug candidates during and after the drug development process is mainly attributed to poor pharmacokinetic properties including poor aqueous solubility, low permeation, short duration of action, and metabolism by the first-pass effect. Consequently, this has led the researchers to study the role of the drug candidate’s pharmacokinetics in the early stages of the drug discovery and development process. 

One of the strategies to improve the pharmacokinetics profile for a drug is via utilizing the prodrug approach which is intended to overcome physicochemical, biological and organoleptic barriers of some of the currently marketed drugs suffering low bioavailability and patient incompliance. The high rate of success seen in recent years using the prodrug approach has encouraged the scientific community to pursue this well-established strategy to develop new therapeutic entities that enjoy better clinical profiles than their parent drugs. 

Altering the physicochemical properties of a certain drug has the potential to change its absorption, distribution, metabolism, and elimination (ADME). For this to be successful, a full understanding of the physicochemical and biological behavior of the parent drug must be a prerequisite. The use of tissue’s *in vitro*–*in vivo* data and *in silico* or computational predictions using quantitative structure- activity relationship (QSAR) or molecular modelling is a successful tool to gain a comprehensive insight into such field. Furthermore, utilizing molecular biology, computational chemistry and data obtained from enzymes and transporters have accelerated the establishment of a new era of prodrugs called ‘targeted drugs’. Consequently, scientists have switched from their traditional methods in synthesizing classical prodrugs and commenced utilizing the targeted prodrugs approach. The outcome of this trend has resulted in the development of new prodrugs with better therapeutic profiles than their corresponding parent drugs.

We believe that the use of the targeted prodrug approach will be facilitated in the coming few years and it is expected that the percentage of prodrugs in the medicines market will to exceed 20%. Using molecular orbital (semi-empirical, *ab initio* and DFT) and molecular mechanics methods along with or without an x-ray and spectroscopic analysis of enzymes and transporter is the cornerstone for the design of more efficient drugs. Most of the prodrugs described in this review were synthesized based on the researchers’ chemical or/and biochemical knowledge gained without the use of computational data. However, to make more successful and effective prodrugs, there is a pressing need for a computational design before any wet chemistry to take place.

During the last decade, we have been studying the mechanisms of some intramolecular processes aiming to understand how enzymes catalyze biochemical transformations. The goal was to identify a computational method that provides correlations between experimental reaction rates and calculated kinetic and thermodynamic values and uses the resulting correlations equations for the design of new prodrugs. Using DFT and MM methods we have explored the mechanisms of several intramolecular processes including Bruice’s cyclization of dicarboxylic semiesters and Kirby’s acid-catalyzed hydrolysis of N-alkylmaleamic acids and found linear correlations between calculated and experimental reactions rates. Based on the resulting equations we have designed and synthesized some novel prodrugs such as dopamine prodrugs to treat Parkinson’s disease, gabapentin prodrugs as an anticonvulsant medication, tranexamic acid prodrugs to treat bleeding conditions, aza-nucleosides prodrugs for the treatment for myelodysplastic syndromes, atovaquone prodrugs as an anti-malarial agent. Additionally, we have invested this approach in masking the bitter sensation of commonly used drugs such as the pain killer paracetamol. In the prodrugs mentioned above, the amine or hydroxyl group in the parent drug was linked to a promoiety such that that the prodrug undergoes an intramolecular reaction to yield the parent drug and a non-toxic promoiety upon its exposure to physiological medium by rate that is only determined on the chemistry of the inactive promoiety [1,2,54].

Based on the above, an efficient design based on understanding the chemistry and biochemistry of enzymes and transporters accompanied with the use of appropriate computational methods is a necessary step for invoking successful prodrugs. 

Toxicity and cytotoxicity of the linker (promoiety) and prodrug must be conducted during the preclinical phase. Moreover, the use of directed enzyme prodrug therapy (DEPT) strategy which employs the design of artificial enzymes to activate prodrugs at specific sites should be widening. Entities to be utilized in DEPT can be directed at genes, antibodies, viruses, and clostridia. The use of this strategy in chemotherapy can significantly improve the clinical profile of the drug and tolerability of the treatment.

It is worth noting, that the utilization of albumin as a protein carrier to cancer cells has a great potential as seen in the case of aldoxorubicin which has emerged as a successful attempt at exploiting tumor accumulation of albumin as well as the acidic environment of solid tumors. If this strategy is succeeded, it could potentially be exploited in the delivery of many anticancer agents into tumors.

In conclusion, the prodrug strategy remains a viable and effective method of making new active entities. This can be incurred by taking into account recently approved prodrugs and the scan of clinical trials conducted during 2013-2018.
